# Do Musicians Have Better Mnemonic and Executive Performance Than Actors? Influence of Regular Musical or Theater Practice in Adults and in the Elderly

**DOI:** 10.3389/fnhum.2020.557642

**Published:** 2020-09-15

**Authors:** Mathilde Groussard, Renaud Coppalle, Thomas Hinault, Hervé Platel

**Affiliations:** UNICAEN, PSL Research University, EPHE, INSERM, U1077, CHU de Caen, Cyceron, Neuropsychologie et Imagerie de la Mémoire Humaine, Normandie Université, Caen, France

**Keywords:** music, theater, practice, cognition, aging, lifespan

## Abstract

The effects of musical practice on cognition are well established yet rarely compared with other kinds of artistic training or expertise. This study aims to compare the possible effect of musical and theater regular practice on cognition across the lifespan. Both of these artistic activities require many hours of individual or collective training in order to reach an advanced level. This process requires the interaction between higher-order cognitive functions and several sensory modalities (auditory, verbal, visual and motor), as well as regular learning of new pieces. This study included participants with musical or theater practice, and healthy controls matched for age (18–84 years old) and education. The objective was to determine whether specific practice in these activities had an effect on cognition across the lifespan, and a protective influence against undesirable cognitive outcomes associated with aging. All participants underwent a battery of cognitive tasks that evaluated processing speed, executive function, fluency, working memory, verbal and visual long-term memories, and non-verbal reasoning abilities. Results showed that music and theater artistic practices were strongly associated with cognitive enhancements. Participants with musical practice were better in executive functioning, working memory and non-verbal reasoning, whereas participants with regular acting practice had better long-term verbal memory and fluency performance. Thus, taken together, results suggest a differential effect of these artistic practices on cognition across the lifespan. Advanced age did not seem to reduce the benefit, so future studies should focus on the hypothetical protective effects of artistic practice against cognitive decline.

## Introduction

Without a doubt, musical practice has become a model for the study of neuroplasticity in cognitive neuroscience over the past 20 years (Altenmüller, 2008; Schlaug, 2015). It is now accepted that musical expertise leads to cerebral reorganizations resulting in changes in the brain anatomy of regions engaged during formal music learning, such as motor (Wan and Schlaug, 2010), auditory perception (Parbery-Clark et al., 2013; Bidelman and Alain, 2015; Zendel et al., 2019) and memory areas (Groussard et al., 2010, 2014; Fauvel et al., 2013). Musical practice also influences cognitive functioning, involving better performance on tasks that directly call upon skills explicitly learned during formal music learning (*near transfers*) but also with an effect on general cognitive functions (*far transfers)* in musicians (Fauvel et al., 2013; Schellenberg and Weiss, 2013; Schlaug, 2015). Studies reported better performance for musicians compared to non-musicians mainly in executive functioning, notably working memory, flexibility and verbal fluency (Degé et al., 2011; Criscuolo et al., 2019).

Some authors suggested that beyond musical practice, an active, socially engaged, mentally and physically stimulating lifestyle can also have a positive effect on cognitive functioning (Jung et al., 2017). Brain activity and structure are shaped by experience throughout the lifespan, even at an old age. This plasticity has often been demonstrated after long and intensive trainings, where performance in trained activity improves after practice and leads to the building of a cognitive reserve that could explain the interindividual variability regarding aging (Stern, 2009; Chan et al., 2018). This suggests that higher cognitive reserve is associated with compensatory adjustment and could slow down age-related cognitive decline (Kalpouzos et al., 2008; Hinault and Lemaire, 2020). Different factors influence the variability of this reserve among subjects, including levels of education and general lifestyle (diet and physical fitness), but also the quality of social interaction and hobbies (Scarmeas and Stern, 2003).

Like music, theatrical practice is an artistic activity that requires many hours of individual or collective training in order to reach an advanced level. This process requires the interaction between higher-order cognitive functions and several sensory-cognitive modalities (auditory, verbal, visual and motor), as well as the regular learning of new pieces (for musicians see Brown et al., 2015). However, only few studies have investigated the positive effect of artistic activities other than music, such as theater, on cognition in adulthood. While theatrical practice also seems to have potential effect on overall well-being and cognition, its effect on cognitive functions is still poorly understood. To our knowledge, only Noice’s team has conducted a series of studies to specify the effect of theatrical practice on cognitive processes. These studies investigated cognitive changes following short-term theatrical interventions in older adults (Noice and Noice, 1999; Noice et al., 2004, 2014; Banducci et al., 2017 for review). They compared older participants who received theater arts training (*n* = 44), visual arts training (*n* = 44) or no-treatment (controls, *n* = 36) during nine 90-min sessions over a month. The pretest and posttest comparison suggested that the performance of the theater arts intervention group was better than no-intervention group on word recall, listening span and problem-solving tasks. Compared to the visual arts group, the theater arts group performed better on problem solving only (Noice et al., 2004). Recent work from this team further expands these results, as Banducci et al. (2017) compared the cognitive benefit of an active acting program including 86 healthy aging versus 93 participants constituting the control group (history of art classroom) for 4 weeks. A cognitive battery was administered before and after intervention, and again in a 4-month follow-up. The participants of the active acting program benefited most relative to the control group in episodic recall only, with gains still evident up to 4 months after intervention. Both groups were similar in the magnitude of gains in working memory, executive function and processing speed. Due to the scarcity of work on theater practice compared to music training in the literature, it seems necessary to specify the benefits of theatrical regular practice on cognition and to better understand its effect throughout life. Many factors appear to influence an individual’s aging trajectory (Raz and Kennedy, 2009; Hinault and Lemaire, 2020), suggesting that interventions could possibly slow down cognitive decline and promote healthy aging. To this end, various behavioral interventions have been proposed, such as physical activity and cognitive training (Colcombe and Kramer, 2003; Jaeggi et al., 2008; Karbach and Kray, 2009; Fauvel et al., 2013; Sprague et al., 2019 for reviews), and the benefits of arts practices for promoting health have received growing interest. Importantly, while prior studies have undoubtedly shown the association between arts engagement and well-being (Mella et al., 2017; Fancourt and Steptoe, 2019), objective measurement of the specific cognitive benefit associated with repeated and regular art practices like acting or music across the lifespan, have not been carried out to the best of our knowledge.

The literature is consistent with results obtained by the first study performed on the link between the practice of a musical instrument and cognitive functions in elderly subjects (Hanna-Pladdy and MacKay, 2011). These authors observed that elderly musicians outperformed elderly non-musicians on non-verbal working memory, naming and executive function tests. Moreover, their study suggested a correlation between musicians considered to have a high level of expertise (i.e., having at least more than 10 years of practice) and the preservation of cognitive functioning while aging. Contrarily, Mansens et al. (2018) showed that the time spent making music was not the most important criterion with respect to cognitive function compared with other practice characteristics such as current amount of time making music or age of onset of musical practice.

To our knowledge, no study has been carried out among people engaged in theater practice in order to evaluate their cognitive abilities and possible reserve effects. However, it seems logical to think that many years of theater practice could influence cognition and especially memory, as actors have to memorize new texts regularly. Similarly, it seems surprising that actors have never constituted a reference group or a comparison group with musicians. This is probably due to the fact that it is more difficult to define equivalent criteria for the level of expertise for actors whose training and practices are more heterogeneous than for musicians educated in music conservatories.

The main objective of this study was to compare the positive influence of musical and theatrical current and regular practice on cognition. Our goal was first to determine, throughout the lifespan, whether people with current musical and theatrical practice could show cognitive differences, and if the number of years of practice influence these modifications. Second, we aimed to study the effect of musical and theatrical practice on cognition in older adults, in order to assess the specific differences in cognition between actors and musicians while aging (e.g., executives and episodic memory processes).

Participants underwent a battery of cognitive tasks evaluating processing speed, executive functioning, fluency, working memory, verbal and visual long-term memories, and non-verbal reasoning abilities. Considering the literature, the main expectation was that both groups (musicians and actors) would perform higher than control subjects without any intensive and regular leisure activity on tasks evaluating both executive functions and memory. We also expected a specific effect of the type of practice on cognition, with better performance for verbal memory in actors and executive functioning and reasoning in musicians. Moreover, we expected that processes involved in reading scores in musicians would increase their abilities in visuo-spatial memory. We finally expected that these patterns would be maintained in older adults.

## Materials and Methods

### Participants

We recruited three groups of healthy subjects differing only in regular and sustained practice of a specific leisure activity (music; theater, no specific practice for the control group). The dataset was obtained from 146 participants, 50 controls; 50 “musicians” and 46 “actors” matched for age and education. Three participants (1 by group) were excluded because they only partially completed the neuropsychological assessment (Table 1). All participants were native French speakers, with normal hearing and normal or corrected to normal vision without any reported neurological or psychiatric conditions, as assessed by a medical interview. None of them presented signs of cognitive impairment (i.e., two or more scores in two or more cognitive domains below two standard deviations of the norms for their age class). All participants provided informed consent before being tested and all procedures were conducted in agreement with the ethical principles of Declaration of Helsinki.

**TABLE 1:** Demographic data of participants and practice background information for Musicians and Actors.

	Controls	Musicians	Actors	Stats	*p*-value
N of subjects	49	49	45		
Gender (F/M)	30F + 20 M	24F + 26 M	30F + 16 M	*X*^2^ = 3.096	0.213
Age	47.47 ± 17.78 [18–80]	47.84 ± 18.3 [20–83]	41.58 ± 18.13 [18–84]	*F* = 1.748	0.178
Years of education	13.9 ± 2.946 [9–20]	14.73 ± 2.139 [9–19]	13.89 ± 2.648 [7–20]	*F* = 1.689	0.188
Age onset of practice	n.a	13.02 ± 12.82 [2–65]	24.9 ± 15.34 [4–70]	U MW = 506	**<0.001**
Years of practice	n.a	31.51 ± 16.67 [5–65]	17.13 ± 10.82 [4–45]	U MW = 539.5	**<0.001**
Weekly practice hours	n.a	13.03 ± 11.03 [1–49]	10.18 ± 9.83 [2–40]	U MW = 1.318	0.191
Number of exibition/year	n.a	18.14 ± 24.84 [0–100]	14.43 ± 22.14 [0–100]	U MW = 0.76	0.448

Controls were defined as participants who had practiced any leisure activity regularly (more than 4 h/week) associated with formal lessons (physical activity or drawing lesson for examples) and had never taken any formal music or acting lessons, that could neither play nor read music.

Participants were included in “Musicians” group when they reported current and regular practice at the moment of the study for more than 3 years of musical instrument without interruption, more than 4 h/week, and if they had received formal music training. In addition to this, Musicians had to never have practiced theater. Musicians were recruited from several French conservatories or music schools (no self-educated musicians were included). They played various musical instruments (piano, guitar, trumpet, etc.). To study the influence of instrumental practice and avoid confounding effect between singing and instrumental practices (e.g., Mansens et al., 2018) we excluded participants who previously performed choral singing in all groups.

Participants were included in “Actors” group when they reported current and regular acting practice at the moment of the study for more than 3 years without interruption, more than 4 h/week, and if they had taken formal theater lessons. In addition to this, Actors must had never practiced a musical instrument. All Actors were recruited from theater companies or cultural associations which offered acting lessons. Without being professional, they learned new texts on a regular basis and had regular live performances (two per month in average).

Musicians’ and Actors’ background information is provided in Table 1, which includes the age onset of practice, number of years of practice, weekly practice hours, number of exhibitions by year.

We then reduced our sample to individuals who were 50 years and older to study the difference in cognition between older practitioners (Schneider et al., 2019). This sample was composed of 27 Controls, 24 Musicians and 15 Actors (see Table 2).

**TABLE 2:** Demographic data of participants of 50 years and older Practice background information for Musicians and Actors of 50 years and older.

	Controls	Musicians	Actors	Stats	*p*-value
N of subjects	27	24	15		
Gender (F/M)	18F + 9 M	12F + 12 M	9F + 6 M	*X*^2^ = 1.47	0.48
Age	61.1 ± 7.29 [51–80]	63.9 ± 8.81 [50–83]	62.6 ± 9.73 [52–84]	*F* = 0.723	0.489
Years of education	14.5 ± 2.65 [9–19]	14.2 ± 1.99 [9–17]	12.9 ± 2.45 [10–20]	*F* = 2.31	0.108
Age onset of practice	n.a	18.5 ± 16.6 [2–65]	37.4 ± 17 [12–70]	U MW = 69.5	**<0.001**
Years of practice	n.a	43 ± 15 [12–65]	23.7 ± 13.4 [4–45]	U MW = 60.5	**<0.001**
Weekly practice hours	n.a	12.8 ± 11.00 [3.5–49]	10.5 ± 11.8 [3–40]	U MW = 130	0.152
Number of exibition/year	n.a	14.3 ± 18.6 [0–60]	15.9 ± 20.4 [2–80]	U MW = 142	0.27

### Cognitive Functioning

Cognitive functioning was measured using several assessments covering various cognitive domains (Table 3) that are clearly impacted by normal aging (Hedden and Gabrieli, 2004; Salthouse, 2010), the earliest and most concerned being processing speed, working memory (maintenance and manipulation of information during a short period of time), spatial ability, reasoning, and episodic memory (declarative long-term contextual remembering of personal events or information). The entire test battery was administered in a single session, which lasted about 90 min, and took place in a quiet room.

**TABLE 3:** Description of the tests and the dependent variables used in the study.

Cognitive functions assessed	Psychometric tests	Dependent variables (outcomes)
Long-term verbal memory	BEM-144’s 12 words (Signoret, 1991)	Total score of the three trials in learning phase (*BEM Total*)
		Number of word recalled in the delayed recall (*BEM Recall*)
Long-term visual memory	Doors test (Baddeley, 1994)	Number of doors recognized (*Doors Total*)
	Rey-Osterrieth complex figure (Rey, 1959)	Score of redraw fidelity (number of details, their completeness and location) *(Rey Recall)*
Working memory	Forward digit span (Godefroy et al., 2008)	Highest number of digits properly recalled in 2/3 trials *(Digit Span)*
Attentional abilities	d2 Test (Brickenkamp, 1981)	Total number of target symbols correctly identified *(GZ-f)*
Executive control and verbal abilities	Phonemic Fluency task (Cardebat et al., 1990)	Total number of words in 2 min *(Phonemic Fluency)*
	Semantic Fluency task (Cardebat et al., 1990)	Total number of words in 2 min *(Semantic Fluency)*
Processing speed	d2 Test (Brickenkamp, 1981)	Number of items processed *(GZ)*
	Digit-symbol coding subtest (Wechsler, 2000)	Number of correct associations in 2 min *(Codes)*
Non-verbal reasoning	Matrix Reasoning subtests (Wechsler, 2000)	Number of matrices properly completed *(Matrix)*

#### Long-Term Memory

Long-term verbal memory was measured using the 12-word subtest from the Signoret BEM-144 (Signoret, 1991). This verbal memory test consists of learning 12 words during 3 sessions. After every trial, participants are asked to recall as many words as possible. Then participants are distracted by performing a non-verbal task for approximately 7 min. After that, they are asked to recall as many words as possible. We used two scores on the 12-word BEM test: the total score of the three trials to evaluate total learning (BEM Total), and the number of words recalled during the delayed recall (BEM Recall) to assess episodic memory.

Long-term visual memory was evaluated using the Baddeley’s Doors test (Baddeley, 1994). This test is a non-verbal recognition test based on colored photographs of doors composed of two parts (A and B). For each part, 12 doors are shown individually for 3 s. Then, the participants are asked to pick out the one out of the four that had been shown before. The score is the number of correct answers of the two parts combined (Doors Total).

The Rey-Osterrieth complex figure (Rey, 1959) was administered and consists of redrawing an abstract geometrical shape from memory that had been copied 3 min earlier (Rey Recall). The maximum final score is 36. This test is classically used for evaluating of visuospatial constructional ability and visual episodic memory.

#### Executive Functioning

The phonological loop of the working memory, which is the ability to retain verbal information for a short time by mean of mental repetition (Baddeley et al., 1998; Baddeley, 2003), was evaluated using the forward digit span (Godefroy et al., 2008). Participants had to immediately recall series of digits in the order they were presented. The score recorded was the size of the forward digit span with 2 successive correct recalls (Digit Span).

We evaluated visual attention using the d2 Test. This test consists of a paper with 14 rows of 47 interspersed “p” and “d” characters. The participant had to cross out as many “d” with two marks above or below them as possible, in any order (target symbols), and had to jump to the next rows every 15 s. The target symbols are relatively similar to the distractors (a “p” with two marks or a “d” with one or three marks). In this study, we used the overall performance score (GZ-f) corresponding to the total number of target symbols correctly identified (Brickenkamp, 1981).

The phonemic fluency task (Cardebat et al., 1990) was used to measure executive functioning. The participant had to name as many words starting with the letter R as possible in 2 min. The score used is the total number of words in 2 min. The semantic fluency was also proposed, in which participants had to name as many fruits as possible in 2 min. Language processing and semantic memory are most the critical components for this task.

Processing speed was measured using the digit-symbol coding subtest from the Weschler Adult Intelligence Scale (WAIS-III, Wechsler, 2000). In this task, each digit (from 1 to 9) was combined with a specific symbol (example 1/− and 9/=) in the upper row. Participants then had 2 min to complete the number maximum of symbols corresponding to the digits presented in the lower rows. The score was the number of correct associations performed in 2 min (Codes). We also used the number of items processed (Gz) in the d2 test (Brickenkamp, 1981) to evaluate the processing speed.

#### Reasoning

We administered the Matrix Reasoning subtests of the WAIS-III (Wechsler, 2000) to estimate participants’ non-verbal reasoning skills. In this test, participants were presented with an unfinished matrix of drawings, and had to choose the drawing that logically completed the matrix. This task is classically associated with fluid intelligence (Matrix) (Carpenter et al., 1990). We used the number of matrices properly completed as a performance score.

### Procedure and Statistical Analysis

To compare all cognitive variables with each other, scores were standardized. Thus, we transformed all neuropsychological measures into *z*-scores using the mean and standard deviation of the control groups (*n* = 49) as the reference population for each measure due to the lack or poor reliability of French published norms for some assessments. Thus, all variables were on the same scale, with a mean of 0 and a standard deviation of 1 based on the control group. The higher the *z*-score, the better the performance. This allowed comparing every performance on the same normalized scale.

In order to test for group difference among cognitive tests, we performed multivariate analyses of covariance (MANCOVA), with type of practice (Controls; Musicians; Actors) as between-subjects factor, the cognitive test scores (BEM Total, BEM Recall, Doors Total, Rey Recall, Digit Span, Matrix, Codes, d2 GZ, d2 GZ-f, Phonemic Fluency, Semantic Fluency) as dependent variables and age as confounding variable. As we included more than two dependent variables and because they are intercorrelated, we opted for a MANCOVA. This statistical analysis accounts for the relationship between dependent variables (Warne, 2014). To complete the multivariate analyses and examine group differences for each cognitive variable, univariate analyses were performed. A family wise Bonferroni’s correction for multiple comparison analyses was carried out 2-by-2 for every significant test.

To further our exploration of the relationship between musical or theatrical practice and cognitive abilities, we performed a second multivariate analysis on cognitive variables restricted to Musicians and Actors and including Age and Years of practice as variables.

In a separate analysis, the same procedure was implemented reducing the sample to people who were 50 years and older to study the effect of expertise on cognition in older adults.

The sample size was based on a power analysis, conducted in G^∗^Power 3.1. Regarding behavioral interactions between age and cognition, assuming an effect size of Cohen’s *f* = 0.65 [derived from Carey et al. (2015)], an alpha of 0.05, and three groups, we determined that a total sample size of at least 15 individuals per study would provide 95% power to detect the effects.

All the statistical analyses were performed with STATISTICA software. The partial Eta square (η^2^_p_) was utilized to estimate effect size. Results were considered significant at *p* < 0.05.

## Results

### Effect of Expertise on Cognitive Variables in Adults

**FIGURE 1:**
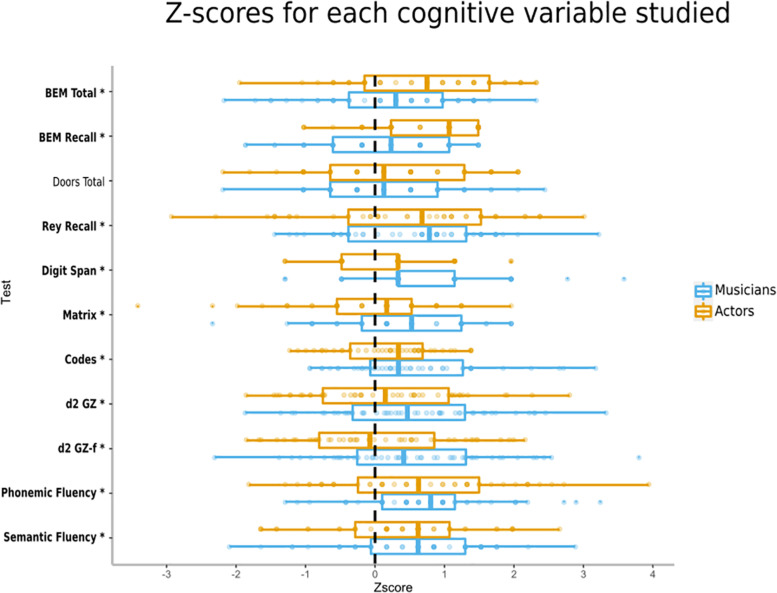
Median, 1st and 3rd quartiles, min and max *z*-scores for each cognitive variable studied. Mean and standard deviation of the control group (*n* = 49) serves as reference population for each measure, as such variables were on the same scale with 0 as the mean and 1 as the standard deviation of the control group. The higher the *z*-score, the better the performance. (^∗^) After a test name indicates a significative difference between Controls and Musicians or Actors.

Results from the multivariate tests on the associations between groups of practice (Controls; Musicians; Actors) and cognitive tests adjusted for age exhibited a significant group effect: Wilks’ Lambda [*F*_(22,258)_ = 3,005, *p* = 0.000015, η^2^_partial_ = 0.204]. Results showed a significant effect of groups of practice with higher values for Musicians relative to Controls for Rey Recall (*p* = 0.024); Codes (*p* = 0.002)*;* d2 GZ (*p* = 0.023); d2 GZ-f (*p* = 0.023); Phonemic Fluency (*p* = 0.002), Semantic Fluency (*p* = 0.019);relative to Actors for Matrix (*p* = 0.006); and compared to both Controls and Actors for Span (*respectively p* = 0.0002; *p* = 0.008). Results also highlighted significantly higher values for Actors than Controls for BEM Total (*p* = 0.002), and Phonemic Fluency (*p* = 0.009), and compared to both Controls and Musicians for BEM Recall (*respectively p* = 0.001; *p* = 0.033) (Table 4 and Figure 1).

**TABLE 4:** Statistical results of MANCOVA for each cognitive variable.

	Controls	Musicians	Actors	Statistics		
				
	Mean ± SD	Mean ± SD	Mean ± SD	*F*	*p*-value	*Post hoc*
BEM Total	25.6734.451	26.8574.21	28.7784.617	**4.450**	**0.0134**	Actors > Controls
BEM Recall	8.4492.39	8.9392.277	10.0671.876	**5.256**	**0.006**	Actors > Controls
						Actors > Musicians
Doors Total	17.6732.593	18.0822.448	18.402.934	0.632	0.533	
Rey Recall	20.8064.716	23.5924.745	23.0676.463	**3.763**	**0.026**	Musicians > Controls
Digit Span	5.5921.29	6.5511.174	5.8220.960	**9.449**	**0.0001**	Musicians > Controls
						Musicians > Actors
Matrix	20.5312.792	21.7352.564	20.2892.928	**5.328**	**0.006**	Musicians > Actors
Codes	69.18417.257	78.77616.37	73.22212.269	**6.906**	**0.001**	Musicians > Controls
d2 GZ	391.30672.378	431.32780.379	401.55679.371	**4.729**	**0.010**	Musicians > Controls
d2 GZ-f	376.87865.827	413.55177.785	382.42271.237	**5.285**	**0.006**	Musicians > Controls
Phonemic Fluency	21.4085.733	25.7555.445	25.207.191	**7.619**	**0.0007**	Musicians > Controls
						Actors > Controls
Semantic Fluency	21.2654.420	23.7964.509	234.661	**3.937**	**0.022**	Musicians > Controls

*SD, standard deviation. Bold values correspond to significant differences between groups.*

The multivariate analysis exhibited an effect of the Age: Wilks’ Lambda [*F*_(11,129)_ = 5.286, *p* = 0.000001], and univariate analyses suggested a significant effect of the Age on BEM Total [*F*_(1,139)_ = 12.49, *p* = 0.0005], BEM Recall [*F*_(1,139)_ = 11.32, *p* = 0.001], Rey Recall [*F*_(1,139)_ = 12.319, *p* = 0.0006], Matrix [*F*_(1,139)_ = 13.85, *p* = 0.0003], Codes [*F*_(1,139)_ = 38.56, *p* < 0.001], d2 GZ [*F*_(1,139)_ = 17.85, *p* = 0.0004], and d2 GZ-f [*F*_(1,139)_ = 20.77, *p* = 0.0001].

### Effect of Years of Practice on Cognitive Variable of Adult Musicians and Actors

The multivariate analysis exhibited a significant effect of groups of practice, Wilks’ Lambda [*F*_(11,80)_ = 4,058, *p* = 0.0001, η^2^_partial_ = 0.358], a significant effect of Age, Wilks’ Lambda [*F*_(11,80)_ = 2,957, *p* = 0.0025, η^2^_partial_ = 0.289] and no effect of Years of practice on cognitive variables Wilks’ Lambda [*F*_(11,80)_ = 1,776, *p* = 0.072, η^2^_partial_ = 0.196]. The univariate analyses on groups of practice confirmed the higher values for Actors relative to Musicians after controlling of Age and Years of practice for BEM Total (*p* = 0.039) and BEM Recall (*p* = 0.0046) and higher values for Musicians relative to Actors for Span (*p* = 0.0023), Matrix (*p* = 0.022), Codes (*p* = 0.0109), d2 GZ (*p* = 0.0404) and d2 GZ-f (*p* = 0.0164).

### Effects of Expertise on Cognitive Variables in Older Adults

**FIGURE 2:**
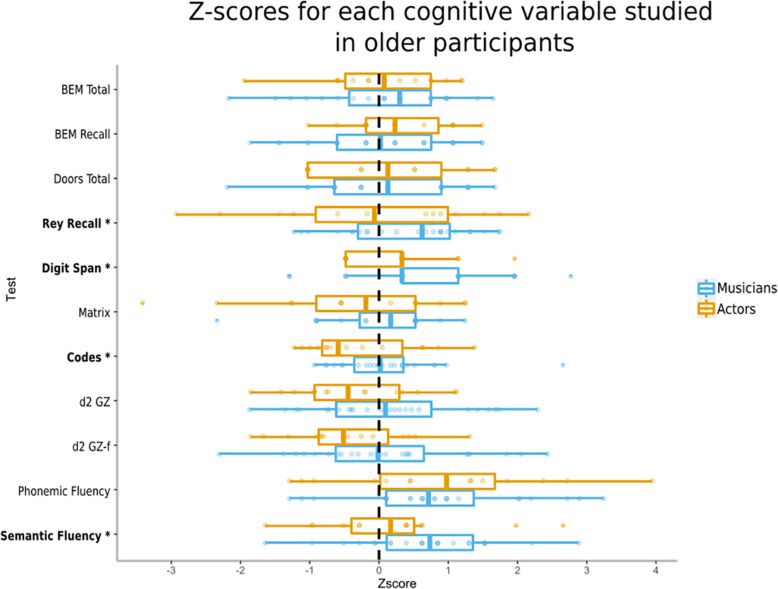
Median, 1st and 3rd quartiles, min and max *z*-scores of the older participants for each cognitive variable studied. Mean and standard deviation of the control group (*n* = 49) serves as reference population for each measure, as such variables were on the same scale with 0 as the mean and 1 as the standard deviation of the control group. The higher the *z*-score, the better the performance. (^∗^) after a test name indicates a significative difference between Controls and Musicians or Actors.

In a separate analysis, we reduced our sample to participant who were 50 years and older to study the effects of expertise on cognitive aging. Results from the multivariate tests that studied the associations between groups of practice (Controls; Musicians; Actors) and the cognitive tests adjusted for Age exhibited a significant group effect: Wilks’ Lambda [*F*_(22,104)_ = 1.732, *p* = 0.0347, η^2^_partial_ = 0.268]. Results of univariate tests showed a significant effect on groups of practice, with significantly higher values in Musicians relative to Controls for Rey Recall (*p* = 0.025), Digit Span (*p* = 0.018), Codes (*p* = 0.026) and Semantic Fluency (*p* = 0.005). No significantly higher values in Actors relative to Controls and difference between Actors and Musicians (Table 5 and Figure 2). The multivariate an effect of age, Wilks’ Lambda [*F*_(11,52)_ = 2.869, *p* = 0.005, η^2^ = 0.378] and univariate analyses suggested a significant effect of age on Doors [*F*_(1,62)_ = 12.82, *p* = 0.0007], Matrix [*F*_(1,62)_ = 5.216, *p* = 0.026], Codes [*F*_(1,62)_ = 14.45, *p* = 0.0003], Phonemic Fluency [*F*_(1,62)_ = 4.614, *p* = 0.036].

**TABLE 5:** Statistical results of MANCOVA limited to older adults.

	Controls	Musicians	Actors	Statistics		
				
	Mean ± SD	Mean ± SD	Mean ± SD	*F*	*p*-value	*Post hoc*
BEM Total	24.84.03	264.20	26.13.81	1.039	0.359	
BEM Recall	82.43	8.462.32	91.69	1.065	0.351	
Doors Total	17.62.61	182.51	17.93.66	0.62	0.541	
Rey Recall	18.84.51	22.64.26	20.87.01	**4.413**	**0.016**	Musicians > Controls
Digit Span	5.441.19	6.381.28	5.870.915	**4.428**	**0.016**	Musicians > Controls
Matrix	19.62.49	20.62.18	19.63.68	1.541	1.756	
Codes	61.315.30	71.113	64.313.9	**5.367**	**0.007**	Musicians > Controls
d2 GZ	38059.60	40179.5	36664.5	1.632	0.204	
d2 GZ-f	36550.70	38376.5	34756.8	1.884	0.161	
Phonemic Fluency	22.66.23	26.36.81	26.68.48	**3.261**	**0.045**	
Semantic Fluency	20.14.67	24.34.44	22.14.83	**5.754**	**0.005**	Musicians > Controls

*SD, standard deviation. Bold values correspond to significant differences between groups.*

### Effect of Years of Practice on Cognitive Variables of Older Musicians and Actors

The multivariate analysis exhibited only a significant effect of Age, Wilks’ Lambda [*F*_(11,25)_ = 2,675, *p* = 0.020, η^2^_partial_ = 0.541]. No effect of Years of practice on cognitive variables Wilks’ Lambda [*F*_(11,25)_ = 1.396, *p* = 0.235, η^2^_partial_ = 0.381] and Groups of practice were observed, Wilks’ Lambda [*F*_(11,25)_ = 0.614, *p* = 0.219, η^2^_partial_ = 0.387].

## Discussion

The aim of the present study was to investigate possible differences in cognition between different art practitioners (Musicians and Actors), and also people without artistic training. Several studies have demonstrated the benefits of musical practice on cerebral activity and cognition, but comparing this practice to another artistic practice such as theater had yet to be done. Our results are consistent with previous works suggesting that adult musicians outperformed control subjects in standardized cognitive tasks (Fauvel et al., 2013; Schlaug, 2015; Sutcliffe et al., 2020) but a lifespan approach was never adopted in anterior studies. In fact, “previous work has focused on younger musicians or older musicians whereas our sample had a very wide age range (from 18 to 84 years), allowing us to study practitioners” cognitive differences throughout the lifespan. We observed a superiority of musicians in long-term visual-spatial memory, working memory, processing speed, executive functioning and non-verbal reasoning. Nevertheless, Musicians did not seem to outperform Controls on verbal episodic memory and visual memory. This result appears consistent with result of a meta-analysis performed by Talamini et al. (2017) on memory. In fact, these authors suggested a small effect size for long-term memory and a possible domain-specific stimuli effect in favor of musical stimuli (Talamini et al., 2017).

Nevertheless, we also found a difference in the theater group on cognition across the lifespan. In line with results of Noice et al. (2004) obtained after a 4-month theatrical intervention, we observed a better long-term verbal memory and verbal fluency in these subjects, compared to controls and musicians. Our results were observed in a large sample of younger to older adults that presented a sustainable practice and intense training. Actors’ better performance in verbal episodic memory is consistent with the abilities developed by them while learning a text and retrieving it during performance. In fact, most actors use mnemotechnical strategies to encode and retrieve their scripts (Noice et al., 2004; Banducci et al., 2017). Strategies were indeed found to improve memory in both young and older adults (e.g., Hinault et al., 2017a,b). However, Actors did not show a difference on executive functioning, working memory and non-verbal reasoning when compared to Control participants. Thus, in future studies it would seem relevant to consider a more detailed evaluation cognition (including strategy use) in order to confirm whether these effects are limited to verbal and memory aspects and do not influence executive processes, or whether self-monitoring abilities are required in theater practice (Nettle, 2006).

This work is the first to statistically compare these two artistic practices, with the same cognitive assessment battery. It highlights for the first time that both musical and theatrical practices could lead to differences in cognition across the lifespan, confirming previous studies on leisure activities, lifestyle and cognition (Hertzog et al., 2008; Reuter-Lorenz and Park, 2014). Furthermore, we observed domain-specific differences, musical practice being associated with better executive functions and reasoning, and theatrical practice with better long-term verbal memory. In fact, Musicians had better performance when compared with Actors on working memory, processing speed, executive functioning, and non-verbal reasoning whereas Actors outperformed Musicians for long-term verbal memory.

In older adults, this pattern seems to be confirmed for musicians, with higher performances on long-term visual-spatial memory, working memory, processing speed and verbal fluency (Hanna-Pladdy and MacKay, 2011; Hanna-Pladdy and Gajewski, 2012; Amer et al., 2013; Fauvel et al., 2014; Mansens et al., 2018; Criscuolo et al., 2019; Ferreri et al., 2019). The EEG study of Moussard et al. (2016) on elderly musicians (currently practicing about 11 h/week) and non-musicians, confirmed a beneficial effect of musical practice on executive control, and highlighted a more anterior distribution of the P3 wave in musicians, suggesting successful functional reorganization in elderly musicians according to the authors. Moreover, longitudinal studies showed that 6 months of piano lessons given to older non-musicians adults could improve working memory and executive functioning (Bugos et al., 2007; Seinfeld et al., 2013). In older actors, Banducci et al. (2017) reported modifications on verbal long term-memory and fluency after a 4-month theatrical intervention in older adults which suggests a cognitive benefit even after a short period of active art practice. While we reported better performances on verbal long-term memory and fluency for Actors compared with Controls, we could not find any significant difference in the elderly for Actors relative to Musicians. These results must to be taken with caution with regard to the small sample size of the older Actors (*n* = 15) and would require further investigation to confirm stronger verbal cognition associated with theatrical practice in aging.

In line with several studies on musicians that suggested no association between practice time and cognitive functions (e.g., Fauvel et al., 2014; Mansens et al., 2018), our results did not reveal any effect of the number of years of practice on assessed cognitive functioning. Thus, having a regular and current practice appears to better explain the cognitive differences we studied rather than years of practice. These results are interesting, as even a short period of practice can lead to an improvement in cognitive performance in adults across the lifespan. There is a growing consensus toward aging brains remaining plastic and consequently involvement in leisure activities such as music or theater remains of significant interest since it is possible to start this type of practice at any age (Noice et al., 2014).

These findings are constrained by several limitations that need to be considered in future research. First, our study is essentially descriptive because of its correlational approach and does not allow us to validate causality. Only future interventional or follow-up studies could confirm these results. Second, we partially evaluated the working memory abilities because working memory updating ability was not assessed, as the digit backward span was not among the cognitive assessment. Future studies could aim at specifying music and theatrical practice differential effects on this cognitive process. Third, musicians and actors differed in their average years of practice, musicians showing a longer practice duration than actors. Although this variable was included as covariate in analyses without significant interactions, it could have explained some of the cognitive differences between our groups. Four, global cognitive functioning was not assessed, but no participant was below the passing score in more than one cognitive measure, in line with preserved overall cognitive performance. Furthermore, although power analyses and previous work support the selected sample size, future studies should investigate cognitive differences between older musicians and actors with a larger sample size.

To conclude, our results suggest that artistic practices can account for different individuals’ aging trajectories (Raz and Kennedy, 2009), and that regular artistic practice could promote the constitution of cognitive and cerebral reserve (Stern, 2009; Stern et al., 2018). Therefore, promoting access to artistic practice could help people maintaining or even improving their cognition, besides the obvious and well-documented interest such activities have on socialization (Belgrave, 2011); well-being (Noice et al., 2004; Castora-Binkley et al., 2010) and developing creativity (Salimpoor and Zatorre, 2013; Reynolds et al., 2016). In line with the evidence reviewed by Sutcliffe et al. (2020) on music training and cognition on aging, our study suggests that musical or theatrical practices, even started late in life, could have an effect on cognitive decline. Ferreira et al. (2015) suggested associations between specific activities and the functioning of individual cognitive domains. Results suggest that cognitive training programs could be individually adjusted to observed cognitive deficits following a neuropsychological assessment, without making it a unique criterion for choosing such activity of course. However, previous works on cognitive interventions for aging and dementia showed mixed results (e.g., Alves, 2013). In future clinical studies it would be interesting to determine whether theatrical interventions could improve language and episodic memory processes for people with deficits in theses domains. Conversely, as both previous works and our results highlight the possible positive effect of musical practice on executive functioning (Mansens et al., 2018; Koshimori and Thaut, 2019; Platel and Groussard, 2020), it seems relevant to specify the effect of musical practice interventions for people with deficits in these processes.

## Data Availability Statement

The raw data supporting the conclusions of this article will be made available by the authors, without undue reservation.

## Ethics Statement

Ethical review and approval was not required for the study on human participants in accordance with the local legislation and institutional requirements. The patients/participants provided their written informed consent to participate in this study.

## Author Contributions

MG and HP conceived the study and coordinated the collect of data. MG and TH performed the statistical analysis. MG wrote the first draft of the manuscript. RC, TH, and HP revised the different version of the manuscript. All the authors approved the submitted version.

## Conflict of Interest

The authors declare that the research was conducted in the absence of any commercial or financial relationships that could be construed as a potential conflict of interest.
